# Changes in Chinese Adults’ Physical Activity Behavior and Determinants before and during the COVID-19 Pandemic

**DOI:** 10.3390/jcm10143069

**Published:** 2021-07-11

**Authors:** Huan Wang, Lianshi Feng, Yanfeng Zhang, Fuhong Zhang, Jinmei Fu, Mei Wang, Dongming Wu, Qiang Feng, Xinhua Liu, Chaoqun Fan, Jingjing Wang, Weizhen Gao, Daniel J. McDonough, Zan Gao

**Affiliations:** 1 National Fitness Survey Center, China Institute of Sport Science, Beijing 100061, China; wanghuan@ciss.cn (H.W.); zhangyanfeng@ciss.cn (Y.Z.); wangmei@ciss.cn (M.W.); wudongming@ciss.cn (D.W.); fengqiang@ciss.cn (Q.F.); liuxinhua@ciss.cn (X.L.); fanchaoqun@ciss.cn (C.F.); wangjingjing@ciss.cn (J.W.); gaoweizhen92@163.com (W.G.); 2 Sport Science and Technology Center of Ningxia Hui Autonomous Region, Yinchuan 750001, China; tiyukejizhongxin@sina.com; 3 Jiangxi Research Institute of Sports Science, Nanchang 330006, China; fujinmei2021@163.com; 4 School of Kinesiology, University of Minnesota-Twin Cities, Minneapolis, MN 55455, USA; mcdo0785@umn.edu (D.J.M.); gaoz@umn.edu (Z.G.)

**Keywords:** moderate and vigorous physical activity, social support, self-determined motivation, sport skills, sport organization

## Abstract

Purpose: To investigate the changes in Chinese adults’ physical activity (PA) behavior and determinants before and during the COVID-19 pandemic. Method: A total of 1028 adults (aged 19–59 years) were recruited from 127 urban and rural neighborhoods in China using stratified three-stage probability sampling. Data collection was conducted in December 2019 and July 2020. Results: Compared with the data before the pandemic, individuals’ weekly moderate-to-vigorous-intensity PA (MVPA) decreased significantly from 139 min to 120 min, seven months after the outbreak (*p =* 0.01), with female and rural populations displaying a more significant decrease (*p =* 0.02). Overall, 13.7% of participants met the PA guidelines (World Health Organization) both before and during the pandemic, while 21.8% met the guidelines only before the pandemic and 18.1% increased their PA and met the PA guidelines during the pandemic. A total of 46.4% did not meet the PA guidelines before or during the pandemic. Determinants of PA behavior change before and during the pandemic included sports skills, self-determined motivation and support from sports organizations. Conclusions: The Chinese adults’ PA levels decreased significantly from before to during the COVID-19 pandemic, particularly among the female population. It is suggested that the enhancement of self-determined motivation, improvement of sport skills, and support from sports organizations might be effective in facilitating individuals’ engagement in PA during the pandemic.

## 1. Introduction

The relationships between individuals’ physical activity (PA) and health outcomes have consistently been an important focus in the field of public health promotion. It is well-documented that insufficient PA is a key risk factor for the incidence of cardiovascular diseases, hypertension, and diabetes, among other chronic diseases [1]. As known, regular participation in PA is an effective way to maintain or improve health-related physical fitness [2]. However, the sudden emergence of the Coronavirus-2019 (COVID-19) pandemic has affected individuals habitual PA behaviors around the world and has led to a decline in global PA levels, which has subsequently increased the risk of developing chronic diseases [3,4]. Researchers in Italy observed an average reduction of 882 MET min/week in PA one to two months after the outbreak of COVID-19 [5]. Likewise, one month after the outbreak in Canada, 22.4% of individuals who used to exercise regularly became physically inactive [6]. In England, a follow-up survey was conducted on individuals’ PA levels from four to eight weeks after the outbreak of the pandemic. Among them, 41% showed a decrease in PA after just four weeks of the outbreak [7]. Similarly, another online survey conducted one month after the outbreak demonstrated that individuals’ average daily PA time decreased by 33.5% [8]. As can be seen, individuals’ PA levels from various countries have been affected to some degree by the severity of the pandemic [9]. 

Although the decline of PA level tended to be a trend during the pandemic, further analysis found that the behavioral changes of the populations can be divided into four groups [10,11], and some people could still adhere to their active behavior and even increase PA. Evidence from the American College of Sports Medicine’s Call to Action shows that people who maintain regular exercise have good immunity function and lower risk of contracting COVID-19 [12]. Another study showed that those who moved from active to inactive had stronger/higher depressive symptoms, loneliness, and stress compared to those who maintained adherence to physical activity guidelines. A German study showed that 31% of Germans reduced their leisure time sport and exercise (LTSE), 27% maintained and 6% intensified their LTSE level, and 36% were not engaged in LTSE [11]. Explorations of the reasons for the change of behavior indicate that the most important factor for reduced LTSE was the lockdown of sports infrastructure. Moreover, adherence to exercise is complex and involves a high level of intrinsic motivation. However, the question of which determinants contribute to becoming physically active has remained unanswered.

According to the Knowledge Attitude and Practice Model [13], individuals’ prerequisite to engage in PA is to have a certain level of knowledge about exercise and the identification of the values of exercise. However, whether an individual’s perceptions or behavioral intentions can be transformed into actual behavior is affected not only by external environments but also one’s personal factors, such as desire and motivation, which in turn affect their PA engagement and maintenance [14]. During the outbreak of COVID-19 in 2020, how individuals’ motivation and external environments affected their participation and maintenance of PA has not been explored in depth. Previous studies have empirically examined the relationship between self-determined motivation and health behavior changes in clinical populations [15,16], with the findings usually suggesting increased levels of intrinsic motivation to be the core driver for individuals to overcome perceived PA barriers and maintain sufficient levels of PA participation.

The purpose of this study, therefore, was to examine the changes in Chinese adults’ exercise attitudes, motivation, and PA behavior before and during the pandemic by gender and urban/rural geographic locations. In addition, we aimed at investigating the related PA determinants of different types of behavior changes with the goal of offering practical implications to facilitate Chinese adults’ resumption of PA engagement to a sufficient PA level as soon as possible. Based on the literature and recent studies, we hypothesized that Chinese adults’ exercise attitudes and PA behavior would decline during the pandemic in general, but that there would be differences among different exercise groups. We also hypothesized that individuals’ intrinsic motivation, sports skills and external environments would emerge as the important determinants of the adults’ PA levels during the pandemic and beyond. 

## 2. Materials and Methods

### 2.1. Study Setting and Participants

The current study was part of the National Fitness Status Survey (NFSS) conducted by the General Administration of Sport of China. The purpose of NFSS was to examine the PA behaviors among Chinese adult residents. It was launched in the year of 2000 with the follow-up survey being conducted about every 5 or 6 years. The 2020 survey was the fourth wave of NFSS. The samples were taken from 31 provinces by using the stratified random sampling procedure involving three stages. The inclusion criteria of the sample were individuals who had lived in the designated local study areas for at least seven months. Exclusion criteria were cognitive and/or language impairment.

**Figure 1:**
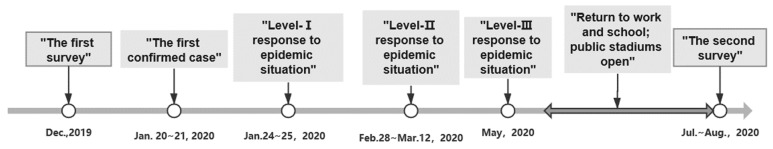
Flow chart of the survey distribution process. Notes: standards for response levels of Chinese public health incidents related to COVID-19: level I: in case of a particularly important public health emergency, the provincial headquarters shall organize and coordinate emergency response work within the administrative area in accordance with the decision-making and unified command of the State Council. Level II: in case of an important public health emergency, the people’s governments at all levels of the incident shall organize and coordinate corresponding emergency agencies to carry out emergency response in accordance with the unified deployment of provincial headquarters. Level III: in case of a relatively important public health emergency, the relevant emergency command agencies of cities above prefecture level shall organize and coordinate relevant emergency response work.

The survey was initiated in December of 2019 and was scheduled to be completed by the end of March 2020. However, on 21 January 2020, the first confirmed cases of COVID-19 in some provinces were identified and the survey collection was suspended. Following this, on 24 January 2020, the local government set the response level of the pandemic to level I (see the notes of Figure 1 for details), and as cases declined with time, the response level was downgraded to levels II and III, respectively. Once the decline of COVID-19 cases was stable, the study resumed from July 2020 to August 2020. The same questionnaire was used to conduct the second survey for the same participants to explore the impact of the pandemic on participants’ habitual PA behaviors. The study flow chart is shown in Figure 1.

Participants in this study were adult residents from four provinces in different regions in China aged 19–59 years. A total of 1531 participants from 14 counties and 127 neighborhoods completed the investigation before the COVID-19 outbreak. Seven months after the outbreak of the pandemic and after the lockdown was lifted, a second survey was conducted among participants who completed the pre-epidemic survey. The number of people willing to participate in the second survey was 1028. Full ethical approval was obtained from the China Institute of Sport Science, Beijing, China (CISS-2019-10-29). All participants provided written informed consent. 

### 2.2. Procedures

Face-to-face interviews were conducted in neighborhoods throughout the designated study locations. The interviewer who had received the training before would ask questions and input the data into the computing platform. The NFSS questionnaire used in this study was compiled by the China Institute of Sports Science [17]. Each interview lasted approximately one hour per participant.

### 2.3. Measures

The questionnaire was consisted of nine parts, including exercise-related knowledge, perception of exercise benefits, attitude towards exercise value, the number of sport skills possessed, physical activities, exercise-related motivation, participation in sports organizations, outdoor walking and cycling environments, and outdoor sanitation. Motivation and PA survey items were from validated international scales [18,19,20,21,22]. Other questionnaire items followed the previous national survey questions [23,24]. Specific items and scoring methods are shown in Table 1 below.

Self-determined motivation was measured by the adapted Behavioral Regulations in Exercise Questionnaire-2 (BREQ-2) survey. The reliability and validity of the questionnaire has been previously demonstrated in adults [18,19].

The BREQ-2 evaluates the three levels of motivation, progressing from amotivation to extrinsic motivation and then to intrinsic motivation. Furthermore, it is divided into six regulation stages, including amotivation, external regulation, introjected regulation, identified regulation, integrated regulation and intrinsic regulation. Specific topics include: “I exercise because others say I should exercise” (extrinsic regulation), “I feel guilty if I don’t exercise” (introjected regulation), “I value the benefits of exercise” (identified regulation), “I exercise because it’s fun” (intrinsic motivation), and “I don’t think I have to exercise” (amotivation). The scale uses a five-point Likert Scale with scores ranging from 1 to 5: “1” represents “not at all true for me”, “5” represents “very true for me”, and “2” to “4” represent different degrees between “not at all true for me” and “very true”. In this survey, three levels of motivation assessment were used in scoring.

Participants’ PA levels were assessed using the modified International Physical Activity Questionnaire Short Form (IPAQ-SF, Chinese version, which was translated by Qu) [20,21]. The IPAQ-SF has been observed to be an acceptable measurement tool in Chinese populations with the Cronbach’s alpha of 0.76 [22]. In detail, participants were asked to recall the number of days and minutes per day spent in moderate- and/or vigorous-intensity PA, and from these data, we calculated participants’ moderate-to-vigorous intensities PA (MVPA). Specifically, the participants’ weekly PA time (frequency multiplied by time) of moderate and vigorous intensity PA was calculated, respectively, and then the MVPA total time was obtained by summing the two intensities. With regard to the criteria for achieving the World Health Organization’s guidelines for PA in adults, a minimum of 150 min MVPA per week was required [25]. Participants were classified into one of four PA groups, which was based on whether or not the MVPA guidelines were achieved before and during the pandemic: group 1 (achieved/achieved, participants who achieved the MVPA guidelines both before and during the pandemic); group 2 (achieved/ unachieved, participants who achieved the MVPA guidelines only before the pandemic); group 3 (unachieved/achieved, participants who achieved the MVPA guidelines only during the pandemic); and group 4 (unachieved/ unachieved, participants who did not achieve the MVPA guidelines before or during the pandemic).

The China Institute of Sport Science launched two rounds of expert opinion consultation. The first round of the expert survey passed the five-point Likert scale to evaluate the importance of the indicators, and the preliminary screening of the indicator system was completed. In the second round of the survey, the adjusted results and questions of the first round of the survey were fed back to the experts, who were asked to continue to evaluate the importance of the revised index evaluation system to determine the final evaluation index system. The Cronbach’s alpha coefficient of total questionnaire was 0.81, with 0.78 in the cognition part, 0.84 in the skill part, 0.82 in the motivation part, and 0.80 in the environment part, indicating acceptable internal consistency.

### 2.4. Statistical Analysis

The descriptive statistics (mean and standard deviations) were used to describe numerical variables (e.g., exercise-related knowledge score, attitude towards exercise, number of sport skills, motivation level, MVPA level, outdoor walking and cycling environment score, outdoor sanitary environment score, etc.). Descriptive statistics of frequency and rate data were used for categorical variables (e.g., gender, age, urban and rural dwelling, income level, educational level, occupation, ways to obtain sports information, rate of reaching the PA guidelines, proportion of participation in sports organizations, etc.) We conducted paired-samples *t*-tests to compare mean differences in numeric variables and chi-square tests to compare the differences in rates and proportions before and during the pandemic. 

We used two logistic regressions to analyze the factors affecting PA behavior changes, one among participants who met the PA guidelines before the pandemic (groups 1 and 2) and one among participants who did not achieve the PA guidelines before the pandemic (groups 3 and 4). The first regression model set group 2 as the reference compared with group 1. The second regression model set group 4 as the reference compared with group 3. The dependent variable was whether or not the MVPA guidelines were met during the pandemic. The independent variables included the baseline values and changes in PA-related knowledge, attitudes, expertise, behavior, and environment before and during the pandemic. Meanwhile, gender, age, urban and rural dwelling, income, education level, and employed or not were used as covariates in the analyses and backward stepwise selection was employed to analyze these factors. This was a two-tailed test with the significance level set to 0.05. The data were analyzed with SPSS software 22.0 (IBM Inst., Chicago, IL, USA).

## 3. Results

### 3.1. The Basic Characteristics of the Survey Sample

The demographic and descriptive characteristics of the participants are shown in Table 2. The sample was comprised of 526 (51.2%) men and 502 (48.8%) women. Approximately 53% of the participants were between the ages of 30 and 49 years. Most of the participants were of Han nationality, with a college for professional training degree or below. A total of 65.1% of the participants had a total annual household income of less than 60,000 yuan (Chinese Currency) and 64.4% of the participants had a job. 

### 3.2. Changes in Physical Activity Behaviors

Compared to before the pandemic, the participants’ MVPA significantly decreased seven months after the outbreak, from 139 min per week to 120 min (*p* = 0.01). Correspondingly, the proportion of participants who met the guidelines for MVPA decreased from 36% before the pandemic to 32% during the pandemic. Particularly, females displayed the greatest decrease in MVPA from before the pandemic to during the pandemic as levels dropped from 35% to 27% (see Table 3).

**Figure 2:**
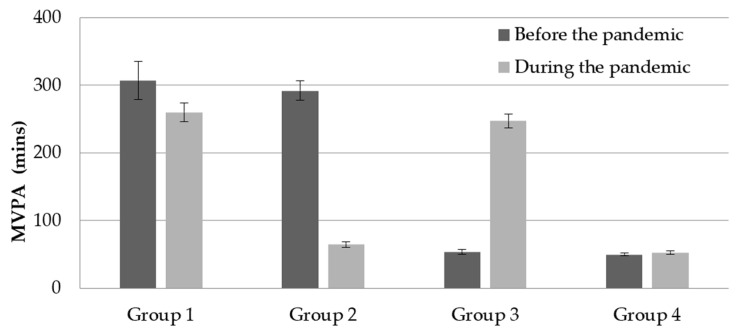
Change in MVPA level among the four groups before and during the pandemic. Notes: group 1 = achieved the MVPA guidelines both before and during the pandemic; group 2 = achieved the MVPA guidelines only before the pandemic; group 3 = achieved the MVPA guidelines only during the pandemic; and group 4 = did not achieve the MVPA guidelines before or during the pandemic.

A total of 13.7% of participants were classified into group 1, with a small variation in MVPA from 307 to 260 mins. A total of 21.8% were in group 2, with a decline in MVPA from 292 to 65 min. A total of 18.1% were in group 3, with an increase in MVPA from 54 to 247 min. Lastly, 46.4% were in group 4, with a small variation of MVPA from 50 to 53 min. The changes in MVPA are shown in Figure 2.

### 3.3. Changes in Knowledge, Attitude, Skills, Motivation and Participation in Sports Organizations

The exercise knowledge score did not statistically significantly differ before and during the pandemic (*p =* 0.16). For the understanding of exercise value and benefits, the overall score significantly decreased from 37.7 points before the pandemic to 36.6 points during the pandemic (*p* < 0.01). Furthermore, the participants’ exercise attitudes and their perceived importance scores significantly increased from 14.7 before the pandemic to 15.4 during the pandemic (*p* < 0.01). Moreover, the number of sport skills mastered after the outbreak increased significantly compared with that of before the pandemic, with an average of 2.9 before the pandemic and 3.7 after the outbreak (*p* < 0.01). In detail, the top-three home exercise methods newly mastered by participants after the outbreak were rope skipping, aerobics or dance, and badminton.

Unsurprisingly, the score of intrinsic and extrinsic motivation significantly decreased (*p* < 0.01). Before the pandemic, 29% of the participants participated in sports organizations, most of whom participated in community sports organizations. However, after the outbreak of the pandemic, this proportion decreased to 24%. For the outdoor walking and cycling environment, 100% of participants reported no significant differences between before and during the pandemic (i.e., the overall score before and during the pandemic was 3.18, *p* = 0.98), while the overall score of the sanitary environment decreased from 3.90 to 3.78 (see Table 4).

Both before and during the pandemic, television broadcasting was the main television viewing channel for participants to obtain sports information but the proportion of participants who obtained information through the Internet increased significantly after the outbreak from 16.7% to 21.7%.

Among the four exercise groups, the motivation level of group 1 and group 2 was higher than that of group 3 and 4 before the pandemic. Moreover, the score of intrinsic motivation in group 1 was higher than that of group 2. There was no difference in extrinsic motivation between two groups. The intrinsic motivation score decreased from 33.0 to 30.9 in group 1 and from 33.4 to 28.7 in group 2 (*p* < 0.05). We observed no statistically significant difference in the motivation level of group 3 (intrinsic motivation *p* = 0.45; extrinsic motivation *p* = 0.27) before and during the pandemic and the motivation level of group 4 was the lowest before the pandemic and decreased significantly after the outbreak of the pandemic (*p* < 0.05; see Table 5 for details). 

The proportion of participation in sports organizations among the four exercise groups is shown in Table 5. The most significant declines in the proportion of participation in sports organizations were in groups 2 and 4, with decreases of 13% and 8%, respectively. Specifically, sports participation in group 1 decreased by 5% while it increased by 5% in group 3.

### 3.4. Factors Related to Change in Exercise Behaviors of Groups 1 and 2

Our logistic regression analysis showed that self-determined intrinsic motivation and the number of sport skills mastered were the most important factors that influenced the change in exercise behaviors of the participants before and during the pandemic. In detail, the odds of participants who had met the MVPA guidelines before the pandemic with more sport skills was increased by 21% (OR = 1.21, 95%CI = 1.06~1.38) compared with those who mastered few sport skills. 

After the outbreak, self-determined intrinsic motivation was positively associated with PA with OR of 1.06 (95%CI = 1.01~1.11). Furthermore, after the outbreak, the greater the self-determined intrinsic motivation and the more types of sport skills mastered, the greater the likelihood of maintaining or exceeding the recommended levels of the World Health Organization’s PA guidelines (see Table 6).

### 3.5. Factors Related to Change in Exercise Behaviors of Groups 3 and 4

The logistic regression analyses also showed that gender, changes in sports organization participation, and mastery of sport skills were important factors influencing changes in participants’ exercise behaviors before and during the pandemic. The odds of the rate of reaching the PA guidelines in males increased by 108% (OR = 2.08, 95%CI = 1.35~3.19) compared with females. The odds of participants who had achieved the PA guidelines before the pandemic with more sport skills was increased by 13% (OR = 1.13, 95%CI = 1.03~1.24) compared with those who mastered few sport skills. Compared with participants who joined sports organizations after the outbreak, the odds of reaching the PA guidelines in participants who quit sports organizations was approximately 56% of the former (OR = 0.56, 95% CI = 0.32~0.98). Overall, males were more likely to increase their PA compared to females (see Table 7).

## 4. Discussion

To the best of our knowledge, this is the first analysis to investigate the changes in adults’ PA attitudes, motivation, environment, and behavior before and during the COVID-19 pandemic. The findings suggested that individuals’ PA attitudes, motivation, and behaviors had all been impacted by the COVID-19 pandemic. In addition, our study examined the association among individuals’ psychological, environmental, and behavioral factors by focusing on four groups whose PA levels changed before and during the pandemic, thus providing a valuable reference for the recovery of PA participation after the outbreak of the pandemic. 

### 4.1. Changes in Exercise Knowledge, Attitudes, Skills, and PA Behaviors before and during the Pandemic

We observed no significant differences in exercise knowledge before and during the pandemic. In general, most participants reported a positive attitude towards and acknowledgement of the importance of PA in which they regarded it as a necessary part of daily living. This observed increase may have resulted from the media stating the importance of being physically active during the COVID-19 quarantine lockdown period, thereby improving participants’ awareness of the benefits and importance of engaging in various PAs. For example, home-based, moderate-intensity exercises may be beneficial to boost the immune system [26] and regulate mood [27]. With a significantly increased percentage of Internet use after the outbreak, data indicated that individuals primarily used the Internet to obtain sports-related information, demonstrating the growing acceptance of online sports.

Seven months after the outbreak of the pandemic, the participant’s types of self-reported exercises increased compared with those before the pandemic. Most of the new skills participants mastered were rope skipping, aerobics or dance, and badminton, primarily due to the fact that these at-home PAs required minimal space and were easy to perform without the use of gymnasiums or other fitness centers. However, the PAs with the highest reported engagement were walking and running. This was in line with findings from other countries. For example, the main PAs among Canadian residents during the pandemic were running, walking, and cycling [6], while in England, individuals’ primary PA during the outbreak were walking, home-based sports, and running [7]. As can be seen, regardless of the country, individuals were more likely to engage in basic movements and try new workouts with lower difficulty and fewer equipment requirements during the COVID-19 pandemic.

The individuals’ MVPA significantly decreased seven months after the outbreak of the pandemic. The findings differed by geographical dwelling and gender. Specifically, participants dwelling in rural provinces experienced significant decreases in MVPA compared to urban-dwelling participants. Females’ MVPA declined significantly more than that of males. Similarly, the proportion of individuals who met the World Health Organization’s weekly PA recommendation declined [25] from 36% before the pandemic to 32% during the pandemic. Indeed, the related research showed that the declines in participants’ PA levels could largely be attributed to the abrupt changes in PA environment and lockdown to exercise equipment brought about by the COVID-19 pandemic [12].

However, our subgroup analysis found that not all the participants experienced a decline in MVPA. That is, PA levels during the pandemic were closely related to the level of before pandemic PA. As previously mentioned, participants were grouped into one of four categories based on whether their PA levels met the PA guidelines before and during the pandemic, respectively: achieved/achieved, achieved/unachieved, unachieved/achieved and unachieved/unachieved. The percentage of participants without changes in PA was 60%, while the rest experienced PA changes before and during the pandemic. This pattern is in line with previous research which employed a survey during the pandemic. A total of 31% of Germans reduced their LTSE, 27% maintained and 6% intensified their LTSE level, and 36% were not engaged in LTSE [11]. 

### 4.2. Participants’ PA Determinants before and during the Pandemic

We divided the four groups into two further subgroups to analyze participants’ PA determinants before and during the pandemic. Prior to the pandemic, MVPA levels of the first subgroup already met or exceeded the minimum PA guidelines established by the World Health Organization. However, only one-third of this group maintained the PA levels during the pandemic, whereas two-thirds of this group experienced a significant decline in MVPA and therefore became the “unachieved” group (i.e., did not achieve the PA guidelines). Using the achieved/unachieved subgroup as the reference, we employed a logistic regression model to analyze the factors contributing to the maintenance of participants’ PA levels. We found that self-determined intrinsic motivation and the number of sport skills acquired during the pandemic were significantly related to the maintenance of PA behaviors and achieving the PA guidelines. In other words, people with relatively higher intrinsic motivation levels and more sports skills were more likely to maintain a higher level of PA during the pandemic. 

Our study found that the participants’ levels of extrinsic motivation and intrinsic motivation showed a downward trend during the pandemic and that there were differences among the four groups on these outcomes. Compared to the achieved/unachieved group, the achieved/achieved group had higher levels of intrinsic motivation for participating in PA. They could be driven by the purpose of “enjoyment, personal values, self-fulfillment through exercise” [28,29,30]. This type of motivation primarily comes from one’s intrinsic needs and desires instead of external factors [14], making individuals more determined to remain physically active. Therefore, we postulate that participants’ higher level of self-determined intrinsic motivation contributed greatly to the maintenance of PA when facing the adverse effects of the pandemic. Other studies have demonstrated the role of self-determined intrinsic motivation in the persistence of exercise rehabilitation in several diseases, although evidence in the context of pandemics is lacking. For example, one study examined the relationship between the self-determined theory and the PA levels of patients with rheumatoid arthritis. It was found that during the intervention, participants’ level of intrinsic motivation predicted MVPA and the degree of persistence in PA after the intervention. In contrast, controlled motivation had no relationship with the maintenance of PA [31]. Similarly, Kinnafick Fetan et al. explored the relationship between the self-determined theory and PA changes in physically inactive employees of a British university [16]. In this study, females were followed for 10 months and their PA behaviors were divided into three categories: participants who were completely inactive, participants who experienced changes in PA (e.g., from starting a PA program to giving up or vice versa), and participants who were consistently physically active. The results indicated that satisfaction with personal demands served as the core motivation to start a PA program and that self-determined intrinsic motivation was an important factor in the maintenance of PA.

In the second subgroup, the individuals did not have sufficient PA levels before the pandemic. Seven months after the outbreak, two-thirds of the individuals remained physically inactive, while one-third had significantly increased their PA levels to meet the PA guidelines. Using the group who did not achieve the PA guidelines before and during the pandemic as the reference group, our logistic regression model was employed to investigate the factors that influenced individuals’ behavior change to become more physically active. We found that the number of acquired sport skills reported by the participants and their participation in sports organizations were significant predictors of PA participation and they were further affected by gender.

Participants who acquired more sport skills during the pandemic and maintained active engagement in organized PAs were more likely to increase their PA levels and meet the PA guidelines. In contrast to the first subgroup, extrinsic and intrinsic motivation did not predict participants’ PA behaviors. In fact, the motivation level of the unachieved/achieved group did not decline during the pandemic and was higher than that of the unachieved/unachieved group. This group had just began participating in PA and the two-way interaction mechanisms of PA and intrinsic motivation had not yet been constructed. Regarding the factors leading to active PA, we observed support from sports organizations to be crucial. Indeed, the self-determined theory asserts that organizational joining is an important way to strengthen extrinsic motivation which requires a continuous process to be transformed into intrinsic motivation [32,33]. Participation in sports organizations, especially during a pandemic, could facilitate encouragement among individuals to engage in PA by imparting skills and experience among members [34]. We also observed the proportion of participants who participated in sports organizations who met the PA guidelines increased during the pandemic, while the proportion of the subgroup that did not meet the PA guidelines decreased. This association was more significant among males compared with females, which may be attributed to the fact that among low active individuals, males were more likely than females to increase their engagement in PA [16].

The common predictor of PA participation in the two subgroups during the pandemic was the number of sport skills acquired. That is, the greater number of sport skills acquired by the participants, the easier it was for them to achieve the World Health Organization’s PA guidelines. Mastering certain sports skills is a prerequisite for engaging in PA [35,36]. Our results demonstrated that, with each additional sport skill acquired, the likelihood of being physically active increased during the pandemic. Our research found that rope skipping, aerobics or dance, and badminton, in addition to traditional walking, running, and cycling, became the newest acquired skills of home workout during the pandemic. Similarly, another study found that fitness skills are an important predictor of individuals’ daily PA behaviors [37]. 

However, there was no association between the improvement of individuals’ exercise knowledge and attitudes and changes in activities behavior during the pandemic, which further demonstrates the discrepancy between exercise knowledge and practice. This discrepancy occurred not only during the pandemic but also in the pre-pandemic era [38]. In 2018, researchers conducted a survey on the PA awareness and behavior of Chinese adults, in which approximately 15% were characterized by the inconsistency between attitudes and behavior. As such, we speculate that whether perceptions can be transformed into behavior is affected not only by external environmental factors, but also by individuals’ personal factors such as exercise perseverance, motivation, and sport skills [39].

This study had two major strengths. First, our research followed the same group of participants at two time points: half a month before the coronavirus outbreak and when the lockdown was lifted with life just returning to a normal, pre-pandemic state. This period presented a good opportunity to explore the impact of the coronavirus pandemic on the respondents’ PA levels. Second, we used three stages of stratified random sampling, thus ensuring the representativeness of the Chinese adults. Despite these strengths, our study had some limitations that we must address. First, because of the sudden outbreak of the pandemic, our sample was relatively small and included just over 1000 participants. Only 67% the participants who took the first wave of survey received a follow-up investigation during the epidemic. However, the missing data were randomly missed after demographic analysis, which had little impact on the representativeness of the sample. Second, our study was conducted over seven months. It began just before the outbreak of the pandemic and ended after quarantine restrictions were lifted. In addition to offering the PA guidelines during the pandemic [40], future longitudinal studies are required to observe and analyze the dynamic change of residents’ behavior during the post-COVID-19 era. 

## 5. Conclusions

This study showed a significant change in participant’ PA after the outbreak of the COVID-19 pandemic. In addition to the impact of the pandemic, the observed inconsistency in PA behaviors among the participants resulted from the combination of personal motivation, the accumulation of sport skills, and support from sports organizations. For individuals who were physically active before the pandemic, the accumulation of sport skills and relatively strong self-determined motivation increased the likelihood of maintenance of adequate PA levels after the outbreak of the COVID-19 pandemic. In contrast, organizational membership and the acquisition of more sports skills could better improve personal PA levels among inactive groups.

## Figures and Tables

**Table 1:** Scoring methods of questionnaire indicators.

Indicators	Assessment	Scoring Method *
Exercise-related knowledge	Appropriate exercise time, exercise intensity, exercise method, related concepts. (e.g., “Early morning is the best time to exercise”, “running should choose a hard and flat ground”, “skipping rope can prevent osteoporosis”, etc.)	A total of 10 questions, the correct answer gets 1 point, and the wrong answer gets 0 point.
Cognition of exercise benefits	Understanding of fitness value, understanding of fitness benefits, etc. (e.g.,” Exercise is good for your health”, “exercise can relieve tension and anxiety”, etc.)	A total of nine questions; each question was divided into one to five points according to the degree of approval
Attitude towards exercise value	Fitness attitude, the status of fitness in the life of participants, etc. (e.g., "For the sports that I like, I am willing to try difficult challenges, etc.)	A total of four questions; each question was divided into one to five points according to the degree of approval
The number of sport skills	32 kinds of fitness skills possible (e.g., Running, playing basketball, playing badminton, swimming, skating, etc.)	One point for each sport skill possessed
Self-determined motivation	Whether to exercise according to their own wishes, whether exercise is performed with confidence. (e.g., “I really enjoy the fun of exercise”, “exercise conforms to my life values”, “it gives me a sense of accomplishment”, etc.)	A total of 12 questions, each question was divided into one to five points according to the answer level
Participation in sports organizations	Eight kinds of sports organizations (e.g., sports organizations established by work units, sports associations for various sports, and sports organizations established by the masses themselves, etc.)	Count the number of options for the organizations, respectively.
Outdoor walking and cycling environments	Participants’ evaluation of walking and cycling environment around their neighborhood	A total of one question, divided into one to five points according to the degree of approval
Outdoor sanitary environment	Participants’ evaluation of the sanitary environment around their neighborhood	A total of one question, divided into one to five points according to the degree of approval

* The performance is evaluated by total score, and higher scores indicate a better performance.

**Table 2:** Demographic information of the participants.

Characteristics	Number of Participants (*N* = 1028) (%)
Gender	
Male	526 (51.2%)
Female	502 (48.8%)
Age	
18–29 years old	146 (14.2%)
30–39 years old	219 (21.3%)
40–49 years old	326 (31.7%)
50–59 years old	337 (32.7%)
Urban and rural	
Urban	669 (65.1%)
Rural	359 (34.9%)
Income (total annual household income)	
Less than 20,000 yuan	220 (21.4%)
20,000–60,000 yuan	449 (43.7%)
Over 60,000 yuan	359 (34.9%)
Educational level	
Primary school or below	235 (22.9%)
Junior middle school	355 (34.5%)
High school, technical secondary school, or technical school	234 (22.8%)
College for professional training	112 (10.9%)
Bachelor’s degree or above	92 (8.9%)
Occupation	
Employed	665 (64.7%)
Unemployed	363 (35.3%)
Marital status	
Unmarried	97 (9.4%)
Married	905 (88.1%)
Divorced or widowed	26 (2.5%)

**Table 3:** Changes in physical activity among different groups before and during the pandemic.

	MVPA (min)		Rate of Reaching the PA Guidelines	
	Before the Pandemic	During the Pandemic	Before the Pandemic	During the Pandemic
Total	138.92 ± 182.64	119.94 ± 123.87 *	36% (33%, 39%)	32% (29%, 35%)
Urban	128.86 ± 164.45	120.08 ± 124.51	33% (29%, 37%)	31% (27%, 35%)
Rural	159.88 ± 214.39	119.71 ± 122.69 *	39% (35%, 43%)	32% (28%, 36%) *
Male	137.95±185.19	124.54 ± 121.47	36% (31%, 41%)	36% (31%, 41%)
Female	140.12 ± 179.96	114.71 ± 126.44 *	35% (30%, 40%)	27% (22%, 32%) *
Group 1	307.98 ± 285.94	260.19 ± 142.77	100%	100%
Group 2	292.36 ± 179.91	65.54 ± 52.46 **	100%	0%
Group 3	53.73 ± 42.89	247.31 ± 118.29 **	0%	100%
Group 4	50.12 ± 41.94	53.23 ± 49.68	0%	0%

* *p* < 0.05 vs. before the pandemic; ** *p* < 0.01 vs. before the pandemic.

**Table 4:** Changes in exercise-related indicators before and during the pandemic.

Indicators	Before the Pandemic	During the Pandemic
Exercise-related knowledge score	5.89 ± 1.80	5.78 ± 1.94
Cognition of exercise benefits	37.66 ± 4.55	36.61 ± 5.51 **
Attitude towards exercise value	14.67 ± 3.16	15.35 ± 3.38 **
Number of sport skills	2.92 ± 1.69	3.69 ± 2.27 **
Home sport skills during the pandemic		
Rope skipping	——	83 (8.1%)
Aerobics, dance	——	69 (6.7%)
Badminton	——	29 (2.8%)
Other	——	569 (55.5%)
Nothing	——	278 (27.0%)
Intrinsic motivation	31.61 ± 5.34	29.45 ± 5.63 **
Extrinsic motivation	9.17 ± 2.43	8.29 ± 3.09 **
Proportion of participation in sports organizations	29% (27%, 34%)	24% (22%, 27%) *
Personal organization	72 (7.0%)	69 (6.7%)
Community organization	142 (13.9%)	95 (9.2%)
City organization	84 (8.2%)	78 (7.6%)
No organization	730 (71.0%)	786 (76.4%)
Ways to obtain sport information before the pandemic		
Television broadcasting	496 (48.2%)	299 (29.0%)
Internet	172 (16.7%)	223 (21.7%)
Books and newspapers	160 (15.6%)	178 (17.3%)
Other	100 (9.7%)	62 (6%)
Do not care	100 (9.7%)	266 (25.9%)
Outdoor walking and cycling environment score	3.18 ± 1.37	3.18 ± 1.09
Outdoor sanitary environment score	3.90 ± 0.85	3.78 ± 0.72 **

* *p* < 0.05 vs. before the pandemic; ** *p* < 0.01 vs. before the pandemic.

**Table 5:** Changes in skills, motivation and participation in sports organizations among the four groups before and during the pandemic.

Indicators	Group 1 (*n* = 141, 13.7%)		Group 2 (*n* = 224, 21.8%)		Group 3 (*n* = 186, 18.1%)		Group 4 (*n* = 477, 46.4%)	
	Before the Pandemic	During the Pandemic	Before the Pandemic	During the Pandemic	Before the Pandemic	During the Pandemic	Before the Pandemic	During the Pandemic
Number of sport skills	3.76 ± 1.75	4.71 ± 2.24 **	3.29 ± 1.58	3.55 ± 2.03	2.96 ± 1.54	4.21 ± 2.21 **	2.97 ± 1.58	3.51 ± 2.17 *
Intrinsic motivation	33.00 ± 5.09	30.91 ± 5.41 **	33.40 ± 4.48	28.67 ± 5.83 **	31.23 ± 5.64	30.76 ± 5.52	30.55 ± 5.43	28.81 ± 5.55 **
Extrinsic motivation	9.45 ± 2.73	7.84 ± 2.96 **	9.77 ± 2.34	7.95 ± 2.99 **	8.91 ± 2.44	8.55 ± 3.04	8.93 ± 2.33	8.48 ± 3.19 *
Proportion of participation in sports organizations	35% (26%, 44%)	30% (21%, 39%)	36% (30%, 43%)	23% (17%, 30%) *	34% (25%, 43%)	39% (30%, 48%)	30% (25%, 34%)	22% (17%, 26%) *

* *p* < 0.05 vs. before the pandemic; ** *p* < 0.01 vs. before the pandemic.

**Table 6:** Related factors of exercise behavior change among participants who achieved the guidelines for MVPA before the pandemic.

Model Dependent Variable	B	S.E.	Sig.	OR	OR95% C.I.	
					Lower Limit	Upper Limit
The number of sport skills mastered	0.19	0.07	0.01	1.21	1.06	1.38
Self-determined intrinsic motivation during the pandemic	0.05	0.03	0.03	1.06	1.01	1.11
Constant	−2.83	0.76	<0.01	0.06		

Abbreviations: B: path coefficients; S.E.: standard error; Sig.: significance; Exp(B): odds ratio; C.I.L: lower limit of 95% confidence interval; CI.U: upper limit of 95% confidence interval.

**Table 7:** Related factors of exercise behavior change among participants who did not achieve the PA guidelines before the pandemic.

Model Dependent Variable	B	S.E.	Sig.	OR	OR 95% C.I.	
					Lower Limit	Upper Limit
Gender (male)	0.73	0.22	<0.01	2.08	1.35	3.19
The number of sport skills mastered	0.13	0.05	<0.01	1.13	1.03	1.24
Sports organization (joined)	REF					
Sports organization (quit)	−0.57	0.28	0.04	0.56	0.32	0.98
Constant	−1.36	0.36	<0.01	0.26		

Abbreviations: B: path coefficients; S.E.: standard error; Sig.: significance; Exp(B): odds ratio; C.I.L: lower limit of 95% confidence interval; CI.U: upper limit of 95% confidence interval; REF: reference group.
